# Analyzing Stabilities of Metal–Organic Frameworks: Correlation of Stability with Node Coordination to Linkers and Degree of Node Metal Hydrolysis

**DOI:** 10.1021/acs.jpcc.4c02105

**Published:** 2024-05-15

**Authors:** Dong Yang, Bruce C. Gates

**Affiliations:** Department of Chemical Engineering, University of California, Davis, Davis, California 95616, United States

## Abstract

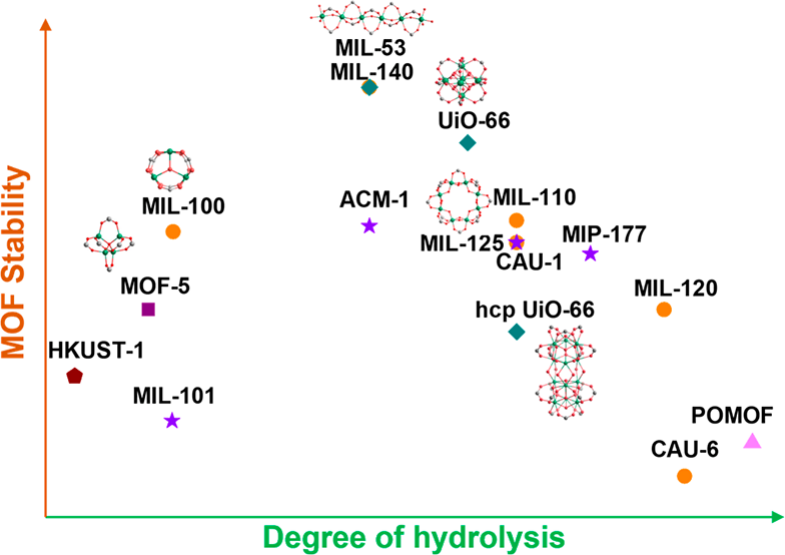

Among the important properties of metal–organic frameworks (MOFs) is stability, which may limit applications, for example, in separations and catalysis. Many MOFs consist of metal oxo cluster nodes connected by carboxylate linkers. Addressing MOF stability, we highlight connections between metal oxo cluster chemistry and MOF node chemistry, including results characterizing Keggin ions and biological clusters. MOF syntheses yield diverse metal oxo cluster node structures, with varying numbers of metal atoms (3–13) and the tendency to form chains. MOF stabilities reflect a balance between the number of node–linker connections and the degree of node hydrolysis. We summarize literature results showing how MOF stability (the temperature of decomposition in air) depends on the degree of hydrolysis/condensation of the node metals, which is correlated to their degree of substitution with linkers. We suggest that this correlation may help guide the discovery of stable new MOFs, and we foresee opportunities for progress in MOF chemistry emerging from progress in metal oxo cluster chemistry.

## INTRODUCTION

The enormous, burgeoning class of porous, crystalline materials known as metal–organic frameworks (MOFs) has a history that can be traced back to 1704 and the chemistry of the pigment Prussian blue. This compound comprises the network structure Fe_4_[Fe(CN)_6_]_3_, which was resolved by Keggin in 1936.^1^ In 1959, Saito took a major step forward, reporting the first compound having a coordination network structure: [Cu(NC–CH_2_–CH_2_–CH_2_–CH_2_–CN)_2_]_*n*_^*n*+^;^2^ this advance is widely regarded as the starting point of metal–organic framework (MOF) chemistry. Saito’s work was extended by Fujita, who in 1994 reported a compound with a two-dimensional coordination network, [Cd(BIPY)_2_](NO_3_)_2_.^3^ This incorporates metal–nitrogen bonds that are too weak to hold the structure together effectively, and a comparable statement pertains to MOFs.

The landmark emergence of MOFs as materials that have high stability (typically measured as resistance to decomposition in air) traces back to several key discoveries, the first reported in 1995 by the group of Yaghi,^4^ who introduced strong metal–carboxylate bonds to stabilize a MOF structure—the MOF is MOF-1, which has the composition [Co(BTC)](NC_5_H_5_)_2_ (BTC is the linker benzene-1,4-dicarboxylate, and Co ions are the nodes to which these bidentate linkers are bonded). The concept was extended and improved by the introduction in early 1999 of Cu_2_(COO)_4_ paddle wheels into a MOF, HKUST-1^5^ (incorporating Cu_3_(BTC)_2_), which was found to have much greater stability than MOF-1 (and is stable at temperatures up to 240 °C in air). A subsequent key advance, made by Yaghi’s group,^6^ was the implementation of metal oxo clusters as MOF nodes, as reported for MOF-5, Zn_4_O(BDC)_3_, in late 1999 (this MOF is stable at 300 °C in air).

MOFs with metal oxo cluster nodes have now grown into a large family incorporating a number of different metal oxo structures and offering a wide range of pore structures and physical properties. Among these are some that are by far the most stable known MOFs. For example, MIL-53 (reported in 2002)^7,8^ and UiO-66 (reported in 2008)^9^ maintain their crystal structures at temperatures up to 500 and 400 °C, respectively, in air.

**Figure 1:**
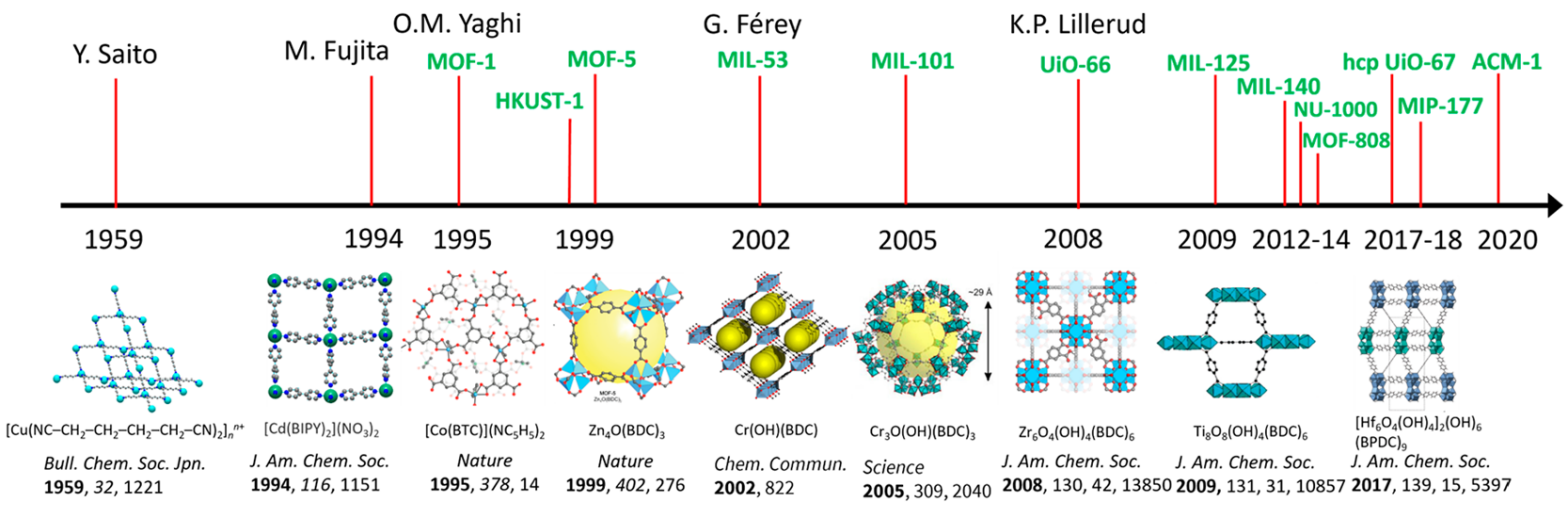
Timeline of advances in chemistry leading to modern developments in MOF chemistry. Structure of [Cu(NC–CH_2_–CH_2_–CH_2_–CH_2_–CN)_2_]_*n*_^*n*+^ adapted with permission from ref (2). Copyright 1959 Oxford University Press. Structure of [Cd(BIPY)_2_](NO_3_)_2_ adapted with permission from ref (3). Structures of MOF-1 ([Co(BTC)](NC_5_H_5_)_2_) and MOF-5 (Zn_4_O(BDC)_3_) reproduced with permission from ref (10). Copyright 2019 John Wiley and Sons. Structure of MIL-53 (Cr(OH)(BDC)) adapted with permission from ref (7). Copyright 2002 Royal Society of Chemistry. Structure of MIL-101 (Cr_3_O(OH)(BDC)_3_) reproduced with permission from ref (11). Copyright 2016 Elsevier. Structure of UiO-66 (Zr_6_O_4_(OH)_4_(BDC)_6_) adapted with permission from ref (9). Structure of MIL-125 (Ti_8_O_8_(OH)_4_(BDC)_6_) reproduced with permission from ref (12). Structure of hcp UiO-67 ([Hf_6_O_4_(OH)_4_]_2_(OH)_6_(BPDC)_9_) reproduced with permission from ref (13).

A timeline of these and other landmark discoveries in MOF science is shown in Figure 1.

The advent of stable MOFs having tailorable textural properties with desirable physical and chemical properties has triggered extensive research on potential applications of these materials, with the primary focus on selective adsorption and separation processes and a secondary focus on catalysis.

## LINKING METAL OXO CHEMISTRY AND MOF CHEMISTRY

Numerous MOFs are synthesized as metal-containing precursors (e.g., zirconium salts) are hydrolyzed, with the formation of metal–oxygen bonds and metal oxo clusters that become MOF nodes. Thus, MOF node formation often involves a combination of hydrolysis and condensation reactions. Linker precursors in the synthesis solutions (e.g., the aforementioned BTC) become bonded to the nodes, often as bidentate ligands that connect the nodes in regular, porous structures that may be highly crystalline.

The chemistry of metal oxo compounds and the chemistry of metal oxide clusters have been invigorated by the emergence of MOFs as a large class of materials that incorporate these structures as nodes. Advances in this chemistry have prompted reconsideration of Pearson’s principle of hard and soft acids and bases,^14^ used to explain MOF stabilities: stable MOFs are formed by combinations of a hard base (e.g., carboxylate as a linker) with a hard acid (e.g., a high-valent metal such as Al, Zr, or Ti in the nodes) or, alternatively, by a soft base (e.g., imidazolate as a linker) with a soft acid (e.g., a divalent metal such as Zn, Cu, or Mn in the nodes).^15,16^ Pearson’s principle has been helpful in guiding the discovery of stable MOF structures. However, for a wide range of metal–ligand combinations, models correlating stability and structure are still lacking; we address this point here.

A large number of MOFs having nodes that are metal oxo clusters have been reported, and it is helpful to classify them. In a review of the evolution of titanium oxo clusters formed by the hydrolysis of Ti(O^*i*^Pr)_4_ (^*i*^Pr is isopropyl) accompanied by incorporation of carboxylate ligands, Schubert introduced a classification of these clusters according to their degree of condensation, *d*_c_ (defined as the number of O^2–^ ions per Ti ion), and the degree of substitution, *d*_s_ (defined as the number of carboxylate groups per Ti ion).^17^ We posit that Schubert’s approach is also valuable for the classification of metal oxo cluster nodes in MOFs—because these, like their counterpart molecular metal oxo carboxylate complexes, are formed in MOF syntheses by the hydrolysis of precursors such as metal alkoxides or metal chlorides as the metals become bonded to carboxylate groups.

**Figure 2:**
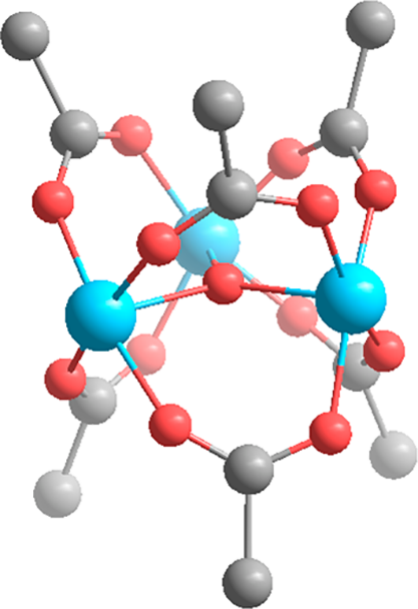
Illustration of the linking of groups to Al_3_O nodes in MIL-100. For simplicity, the bidentate linkers are truncated. Color code: Al, blue; O, red; C, gray.

The degrees of condensation (*d*_c_) of these nodes in MOFs can correspondingly be defined as the number of O^2-^ and OH^-^ groups (mostly μ_2_-O and μ_3_-O; sometimes μ_4_-O) per node metal atom, and then *d*_c_ = *N*_oxygen_/*N*_metal_. For example, in the structure shown in Figure 2, the value of *d*_c_ characterizing the Al_3_O nodes of MIL-100 is 1/3. Terminal node-coordinated ligands such as the common HO^-^–, H_2_O–, and O= are not included in this accounting because they are not part of the metal oxo cluster core.

In the MOFs having carboxylate linkers, the degree of node substitution *d*_s_ is the number *N* of linker carboxylate groups per node metal atom: *N*_linker carboxylate_/*N*_metal_. In the example of MIL-100, *d*_s_ is 6/3 = 2 (Figure 2). Adventitious monocarboxylate ligands such as the commonly observed formates and acetates on the nodes (which arise in many MOF syntheses, for example, from decomposition of *N*,*N*-dimethylformamide (DMF) used as a solvent and from acetic acid used as a modulator) are not included in this accounting because, in contrast to the linkers, they do not contribute to stabilization of the MOF frameworks.

## CORRELATING MOF NODE DEGREE OF SUBSTITUTION, DEGREE OF CONDENSATION, AND MOF STABILITY

**Figure 3:**
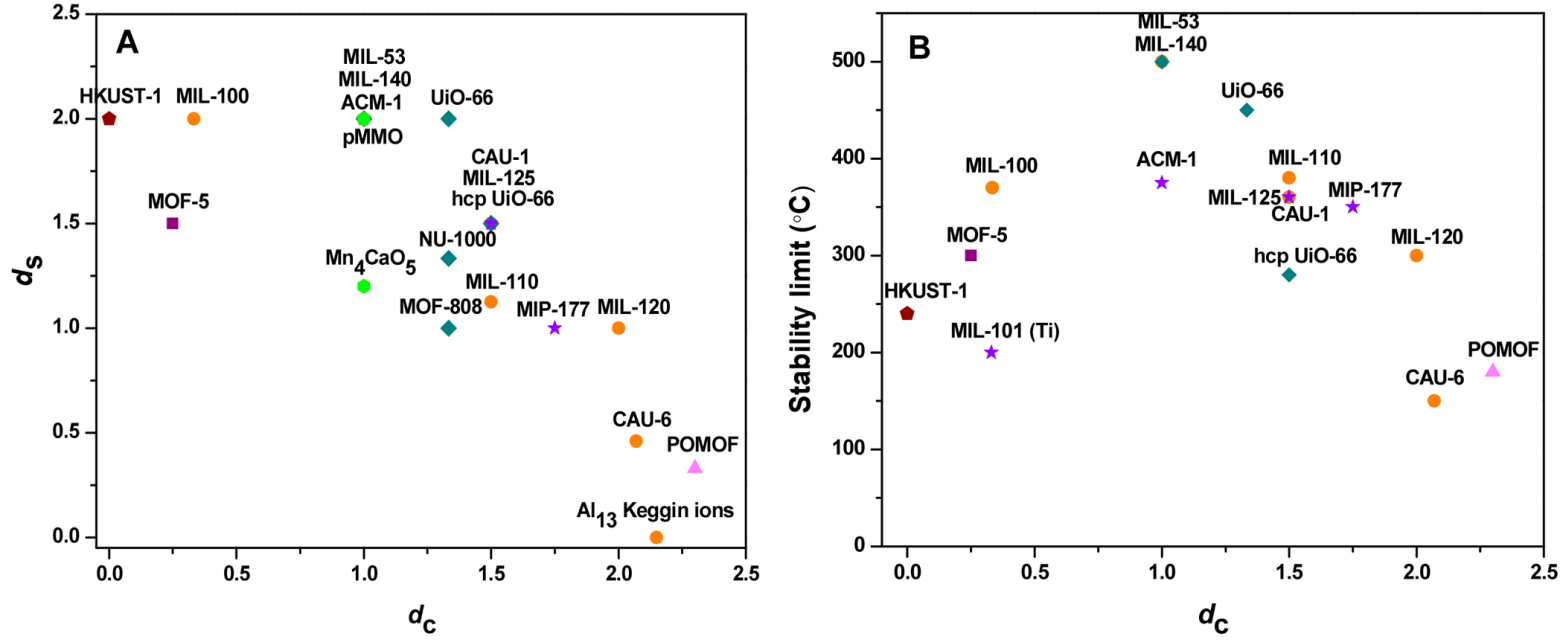
(A) Correlation between degree of node substitution (*d*_s_) and degree of condensation (*d*_c_) of nodes in various MOFs. (B) Correlation between temperature of MOF stability limit (°C) and degree of condensation (*d*_c_) of nodes in various MOFs. The sources of the crystallographic data from which these values were determined are included in Table 1. The α-Al_13_ Keggin ion is used as a reference for fully hydrolyzed precursors. Data characterizing biological clusters, including Fe_2_ clusters in pMMO and Mn_4_CaO_5_ clusters in Photosystem II, are included for comparison. Separate colors are used to distinguish the various metals.

The values of *d*_c_ and *d*_s_ are correlated to each other, as shown by the available data (Table 1 and Figure 3A), which were determined by the reported ideal crystal structures of the MOFs, as established in crystallography experiments and cited in the table.

**Table 1:** Molecular Structures of MOFs with Metal Oxide Cluster Nodes and *d*_s_ and *d*_c_ Values, with Comparisons of Biological Structures

MOF or biological host of metal oxo nodes or pure compound	node or metal oxo cluster	linker^a^	*d*_c_	*d*_s_	temperature determining stability limit in air, °C (method of determination)^b^	ref
HKUST-1	[Cu_3_]^6+^	BTC_2_	0	2	240 (TGA, HTSCXRD)	(5)
MOF-5	[Zn_4_O]^6+^	BDC_3_	0.25	1.5	300 (SCXRD)	(6)
MIL-53	[Al(OH)]^2+^	BDC	1	2	500 (TGA, HTPXRD)	(8)
CAU-1	[Al_8_(OH)_12_]^12+^	BDC_6_	1.5	1.5	360 (TGA, HTPXRD)	(18)
MIL-100	[Al_3_O(OH)]^6+^	BTC_2_	0.33	2	370 (HTPXRD)	(19)
MIL-101	[Al_3_O(OH)]^6+^	(BDC-NH_2_)_3_	0.33	2	377 (TGA)	(20)
MIL-110	Al_8_(OH)_12_X_3_^c^	BTC_3_	1.5	1.25	380 (TGA)	(21)
MIL-120	[Al_4_(OH)_8_]^4+^	BTEC	2	1	300 (TGA, HTPXRD)	(22)
CAU-6	[Al_13_(OH)_27_(H_2_O)_6_Cl_6_(C_3_H_7_OH)_6_]^6+^	(BDC-NH_2_)_3_	2.07	0.46	150 (TGA, HTPXRD)	(23)
Al_13_ Keggin ions	[Al_13_O_4_(OH)_24_(H_2_O)_12_]^7+^		2.15	0		(24)
MIL-140A	[ZrO]^2+^	BDC	1	2	500 (TGA, HTPXRD)	(25)
UiO-66	[Zr_6_O_4_(OH)_4_]^12+^	BDC_6_	1.33	2	400–450 (TGA, PXRD)	(9, 26)
NU-1000	[Zr_6_O_4_(OH)_4_]^12+^	TBAPy_2_	1.33	1.33	350 (HTPXRD)	(27)
MOF-808	[Zr_6_O_4_(OH)_4_]^12+^	BTC_2_	1.33	1	250 (HTPXRD)	(28)
hcp UiO-66	[(Zr_6_O_4_(OH)_4_)_2_(OH)_6_]^18+^	BDC_9_	1.5	1.5	280 (HTPXRD)	(29)
MIL-101	[Ti_3_O(X)]^6+ ^c	(BDC-NH_2_)_3_	0.33	2	200 (TGA, HTPXRD)	(30)
ACM-1	([TiO]^2+^	(TBAPy)_0.5_	1	2	375 (TGA)	(31)
MIL-125	[Ti_8_O_8_(OH)_4_]^12+^	BDC_6_	1.5	1.5	360 (TGA, HTPXRD)	(12)
MIP-177	[Ti_12_O_15_(OH)_6_(H_2_O)_6_]^12+^	MDIP_3_	1.75	1	350 (TGA, HTPXRD)	(32)
POMOF	[PMo_12_O_35_(OH)_5_{La(H_2_O)_3_}]^6+^44H_2_O	BTC_2_	3.3	0.33	180 (TGA, PXRD)	(33)
Photosystem II	[Mn_4_CaO_5_]^6+^	(COO)_6_	1	1.2		(34)
pMMO	[Fe_2_(OH)_2_]^4+^	(COO)_4_	1	2		(35)

a The abbreviations designating the linkers are the following: BDC^2-^, benzene-1,4-dicarboxylate; BTC^3-^, benzene-1,3,5-tricarboxylate; (BDC-NH_2_)^2-^, 2-aminobenzene-1,4-dicarboxylate; BTEC^4-^, benzene-1,2,4,5-tetracarboxylate; TBAPy^4-^, tetrakis(*p*-benzoate)pyrene; MDIP^4-^, 3,3′,5,5′-tetracarboxydiphenylmethane; COO^-^ represents carboxyl ligands from amino acids.

b Stability measured in air by thermal gravimetric analysis (TGA), high-temperature single-crystal X-ray diffractometry (HTSCXRD), and high- temperature powder X-ray diffractometry (HTPXRD); the materials were heated offline when stability was determined by single crystal X-ray diffractometry (SCXRD) or powder X-ray diffractometry (PXRD).

c X refers to undetermined negatively charged inorganic ligands, which may be OH^-^, NO_3_^-^, or Cl^-^, depending on the precursor.

Some early MOFs contain nodes formed from nonhydrolyzed or only partially hydrolyzed precursors, exemplified by MOF-1,^4^ HKUST-1,^5^ and MOF-5.^6^ Consequently, these MOFs have a strong tendency to react with water (to be further hydrolyzed); therefore, they lack stability under moist conditions. MOFs having nodes that have undergone higher degrees of hydrolysis are more stable, with the peak in stability appearing for MIL-53^8^ and UiO-66.^9^ Further increases in the degree of hydrolysis of the nodes, however, lead to decreased MOF stability because they come at the expense of node–linker bonds that have already formed and hold the MOF framework together. Thus, the general trend is that *d*_s_ and *d*_c_ are inversely correlated, so that the greater the degree of hydrolysis of the node precursors and the resultant metal oxide cluster nodes, the fewer the carboxylate linkers bonded to the clusters (Figure 3A)—to the detriment of the MOF stability.

Not surprisingly, higher degrees of hydrolysis often lead to higher degrees of condensation and thus higher-nuclearity metal oxo clusters. For example, increasing *d*_c_ values are observed as Al-containing clusters grow from Al_3_O in MIL-100,^36,37^ to Al_8_(OH)_12_ in CAU-1,^18,38^ and further to Al_13_(OH)_27_(H_2_O)_6_Cl_6_ in CAU-6^23^ (here we are not considering the MOFs with nodes that are chains). Similar trends are evident for Zr- and Ti-containing MOFs (Table 1). The maximum numbers of metal atoms in metal oxo cluster nodes of MOFs are normally less than 13 (not considering the nodes that are chains)— few enough to leave sufficient bonding sites for organic linkers to hold the MOF frameworks together.

## OXYGEN IN MOF NODE SYNTHESES

In MOF syntheses, the formation of metal oxo cluster nodes requires the presence of oxygen sources, for example, H_2_O or H_2_O_2_, in compounds that facilitate the hydrolysis of the metal-containing precursors, such as ZrCl_4_. The oxygen-containing reagents are either added initially to the synthesis mixtures or formed by the reactions of precursors with solvents. For example, the original synthesis of MOF-5 succeeds by the addition of H_2_O_2_ to the synthesis solution of Zn(NO_3_)_2_ and H_2_BDC in DMF/chlorobenzene (combined with the diffusion of triethylamine into the solution).^6^ The synthesis of UiO-66 from ZrCl_4_ and H_2_BDC in DMF requires small amounts of water in the solution to facilitate the formation of the ZrO_4_(OH)_4_ core, leading to the formation of the nodes that contain 6 Zr atoms.^39,40^

In many reported MOF syntheses, the node precursors are hydrates of metal salts, exemplified by ZrOCl_2_·8H_2_O, AlCl_3_·6H_2_O, and Al(NO_3_)·9H_2_O; when these are used, no additional water is needed. For example, the syntheses of NU-1000^41^ and MOF-808^42,43^ proceed from ZrOCl_2_·8H_2_O without added water. Nonetheless, some MOFs are made in the presence of water as a solvent, exemplified by those in the Al-containing MIL family.^44^ Sometimes, the water is formed in situ, as in the synthesis of MIL-125(Ti), whereby the water needed for the formation of Ti_8_O_8_(OH)_4_ nodes is generated in the esterification reaction of Ti(O^*i*^Pr)_4_ with methanol.^12^

Although water is crucial to the synthesis of MOFs that incorporate metal oxo cluster nodes, too much water in the synthesis mixture may be detrimental, leading to overhydrolysis, hindering the growth of MOF crystals and resulting in materials with poor crystallinity. This point is illustrated by observations made during the synthesis of UiO-66.^40^

Another important point about water in MOF synthesis is that it sometimes can be the key component needed to tune the formation of the desired crystalline structure. For example, additional water in the synthesis solution can favor the conversion of the initially formed fcu UiO-66(Hf) (with Hf_6_O_8_ nodes) into hcp UiO-66(Hf) (with Hf_12_O_22_ nodes).^45^

## MOF NODE CHEMISTRY: STABILITY, REACTIVITY, AND THE ROLE OF METALS

The data in Figure 3 show that the Al-containing MOFs span a wider range of *d*_c_ values than the other MOFs, illustrating that a rich and diverse aluminum oxo chemistry has been extended into MOF chemistry. Considering Al_13_ Keggin ions with the composition [Al_13_O_4_(OH)_24_(H_2_O)_12_]^7+^ to be a reference that we describe as fully hydrolyzed,^46,47^ we recognize that some of the MOF metal oxide nodes represent a high degree of hydrolysis, exemplified by the polyoxometalate MOFs (POMOF) CAU-6 and the Mo-containing POMOF.^48^ In Al-containing MOF nodes, as the degree of hydrolysis increases, in the order MIL-100 (370 °C)^19^ < MIL-53 (500 °C)^8,49^ < (CAU-1 (360 °C)^18^ = MIL-110 (380 °C)^21^) < MIL-120 (300 °C)^22^ < CAU-6 (150 °C),^23^ the stability (represented by the temperature at which decomposition occurs, as in Table 1, and shown here in parentheses) shows a clear pattern, first increasing and then decreasing, with the maximum stability observed for MIL-53, with an intermediate *d*_c_ value of 1.0 (Figure 3B).

In contrast, the range of degrees of hydrolysis of Zr-containing MOFs is narrow, with values of *d*_c_ falling only between 1.0 and 1.5 (and increasing in the order MIL-140A^25^ < UiO-66^9^ < hcp UiO-66^29^). The stabilities of these MOFs decrease in the same order (Figure 3B).

To understand the chemistry of these MOFs, it is important to realize the opportunities for tuning the MOF properties by choice of the ligands that are bonded to the nodes, and these include ligands in addition to the linkers. Consider the nodes containing 6 Zr atoms (often approximated as Zr_6_O_4_(OH)_4_ clusters): the node–linker bonding ranges from 12-coordination in UiO-66 (400–450 °C),^9,26^ to 8-coordination in NU-1000 (350 °C),^27^ to only 6-coordination in MOF-808 (250 °C),^28^ where again the temperatures in parentheses represent the stabilities. Thus, in NU-1000 and MOF-808, with their low numbers of linkers per node, there are numerous node sites that can be bonded with ligands other than linkers. These ligands may arise in the syntheses (e.g., adventitious acetate when acetic acid is used as a modulator) or may alternatively result from postsynthesis treatments, such as ligand exchanges. These ligands provide opportunities for tuning MOF reactivity. Commonly encountered examples of nonlinker ligands on the nodes are monocarboxylate, alkoxide, aqua, and hydroxyl.^50−52^ The ligands on UiO-66 nodes have been investigated in some detail, demonstrating a rich chemistry and broad opportunities for manipulating them and the MOF reactivity.^53^ Similarly rich chemistry is anticipated for NU-1000 and MOF-808.

The data thus demonstrate a trade-off: a decrease in the number of carboxylate linkers coordinated to a node (which can be thought of loosely as an increase in node defect density) comes at the cost of MOF stability but at the same time offers greater opportunities for tuning reactivity—and therefore greater potential for applications such as for separations technology and catalysis*—*because these often benefit from optimized reactivity. Optimum reactivity may be dialed in by incorporating node groups that have reactivities that complement each other—and those that complement the reactivities of adsorbates in separations or substrates in catalytic reactions.^54^

The chemistry of MOF nodes is more complex than we have so far represented it to be when the metals in them take on multiple oxidation states. This point is illustrated by the family of Ti-oxo-containing MOFs, which has recently grown rapidly.^55−58^ An early example is MIL-125,^12^ which incorporates a Ti_8_O_8_(OH)_4_ ring structure; later, Ti(III)-MIL-101 was reported,^30,59^ with Ti_3_O nodes (this is air-sensitive), followed recently by MIP-177,^32^ with its highly condensed Ti_12_O_15_(OH)_6_ nodes, and ACM-1,^31^ which incorporates Ti–O–Ti chain nodes. The pattern shown in Figure 3 characterizing these MOFs is similar to that characterizing the Al-containing MOFs: the stability of the Ti-containing MOFs first increases and then decreases with an increasing degree of hydrolysis of the nodes: MIL-101 (200 °C) < ACM-1 (375 °C) < MIL-125 (360 °C) < MIP-177 (350 °C) (with the stabilities again represented by the temperatures shown in parentheses). The most stable Ti-containing MOFs (illustrated by ACM-1) are much less stable than the most stable Zr- and Al-containing MOFs (Figure 3B). Because the stability limits of ACM-1, MIL-125, and MIP-177 are so close to each other, we suggest that the changes in Ti oxidation states (between Ti^3+^ and Ti^4+^) might affect the stability; oxidation of MOF linkers by air might even be catalyzed by the Ti-containing nodes and contribute to the MOF decomposition. There is lots to be learned yet about the mechanisms of decomposition of MOFs and the roles of node metal oxidation states.

Widely investigated MOFs with node metals that have variable oxidation states include those with V, Fe, and Cr. The degree of hydrolysis of nodes containing these metals falls in a narrow range (the corresponding *d*_c_ are between 0.33 and 1.0). They all form M_3_O nodes, in MIL-100, and M–(OH)–M chain nodes, in MIL-53, but reports of MOF nodes with these metals and relatively high degrees of hydrolysis are relatively rare—possibly, we suggest, because the corresponding MOFs might not be stable. The stabilities of the known examples are as follows: MIL-53 (Cr) (375 °C)^60^ > MIL-100 (Cr) (275 °C);^61^ MIL-53 (Fe) (300 °C)^62^ > MIL-100 (Fe) (270 °C);^63^ MIL-47(V) (400 °C)^64^ > MIL-100 (V) (250 °C) (again, with the temperatures in parentheses representing stabilities).^65^ These data characterizing Fe-, V-, and Cr-containing MOFs all indicate the same trend as the data characterizing Al- and Ti-containing MOFs. For example, MIL-53 structures with higher degrees of hydrolysis have higher stabilities than MIL-100 structures, which are characterized by lower degrees of hydrolysis. Moreover, MIL-53 and MIL-100 incorporating Fe, Cr, or V are all less stable than their Al-containing counterparts—again, we suggest, because of their redox properties.

Bolstering the interpretation above about the importance of the metal, the stability data shown in Table 1 for MIL-100(Al) and MIL-101(Al) are very close to each other (370 °C vs 377 °C), thus indicating that the influence of the linkers is not as significant as the influence of the node metals and the degree of hydrolysis.

We reemphasize that the MOF stabilities represented here were all determined by heating the MOFs in air and determining the temperatures at which the MOFs lost structural integrity. The available MOF stability data are limited; reports of systematic investigations of the stabilities of MOFs under conditions of potential applications are still largely lacking. MOFs that have been investigated as catalysts show much less stability in reactions involving strong interactions of reactants or products with the MOF frameworks than in reactions not characterized by such interactions, although there are only few data for comparison. For example, UiO-66 was found to gradually lose its crystallinity under conditions of dehydration of methanol or of ethanol at approximately 250–275 °C—resulting from ester forming reactions involving the alcohols and linker carboxylate groups, which led to disintegration (unzipping) of the MOF frameworks.^66,67^ We have also shown that MOF-808 is much less stable than UiO-66 under conditions of *tert*-butyl alcohol dehydration catalysis at 150 °C, with an equivalent explanation of the MOF disintegration.^54^ MIL-140A has a stability similar to that of UiO-66 under methanol dehydration conditions,^68^ and we have unpublished data showing that hcp UiO-66 is much less stable than UiO-66—as it does not survive methanol dehydration conditions at 200 °C.

The stability trends determined under catalytic alcohol dehydration reaction conditions are in line with the MOF thermal stability data presented above; we doubt whether this comparison has fundamental meaning. Much work is needed to determine and explain stabilities of MOFs under conditions of potential applications.

## COMPARISON WITH NATURE: METAL OXO CLUSTERS IN ENZYMES

The points summarized here extend to nature, exemplified by clusters such as Mn_4_CaO_5_ in Photosystem II,^34,69^ which catalyzes H_2_O oxidation to give O_2_, and the diiron oxo clusters in enzyme pMMO,^35^ which catalyzes methane oxidation to methanol. These biological clusters, like those in MOFs, are bonded to and stabilized by carboxylate ligands (and also a few amine groups, with both kinds of ligands arising from amino acids). Figure 3 shows that these clusters fall in the stable region characterizing the MOFs—note the position of pMMO, which aligns with the most stable MOF. This comparison suggests that the degree of hydrolysis of these bioclusters has been optimized through evolution. (Each of these resolved enzyme structures was in one of the resting states that were detectable and likely more stable than those of working/transition states.)

## OTHER MOF PROPERTIES THAT INFLUENCE STABILITY

We emphasize that although it is beyond the scope of this work, the stability of MOFs is influenced, sometimes strongly, not just by the degree of hydrolysis and the metals in the nodes but also by the functional groups on the organic linkers, the density of defects in the MOF structures, and any impurities bonded to the nodes.^70^ Only few data are available to assess these issues. Thus, considering the number of MOFs represented in the correlations of Figure 3, we are tempted to suggest that the upper limit of MOF stability in air may be roughly 400–500 °C, with the limitation attributable, we suggest, at least in part, to the possibility that the metal-containing nodes may catalyze burning of the organic linkers at such temperatures.

## LINKING CHEMISTRY OF MOF NODES AND CHEMISTRY OF METAL OXIDE CLUSTER CARBOXYLATES

In the past, many metal oxide nodes in MOFs have been borrowed (sometimes inadvertently) from the carboxylate complexes of metal oxide clusters, for example, Zn_4_O(CH_3_COO)_6_^71^ and [Cr_3_O(CH_3_COO)_6_(H_2_O)_3_]Cl·6H_2_O,^72^ which were known long before MOF-5 and MIL-100/MIL-101 were created. Zr_6_O_4_(OH)_4_ nodes of UiO-66 (a MOF first reported in 2008) had earlier been reported as a complex of methyl acrylate (Zr_6_O_4_(OH)_4_(OMc)_12_) in 1997 by Schubert.^73^ Similarly, Zr oxo chain nodes of MIL-140 (dating from 2012) can be viewed as extensions of the Zr_4_O_2_(OMc)_12_ ladder structure that was also reported by Schubert in 1997.^73^ The Zr_12_ node of hcp UiO-66 (reported in 2017) is very similar to the dimeric Zr_6_ clusters reported as [Zr_6_O_4_(OH)_4_(OOCC_2_H_5_)_12_]_2_, again by Schubert in 2006.^74^

Thus, we see the centrality of Schubert’s work as a foundation of MOF chemistry and the connections between the MOF node and linker chemistry. Recent work has reinforced the importance of metal oxide cluster chemistry in the discovery of MOFs: thus, some of the more newly discovered metal oxide clusters (which had not been reported as carboxylate complexes) have now been linked to MOF chemistry. For example, the MOF MIP-177 has Ti_12_O_15_(OH)_6_ nodes,^32^ and the MOF MIL-110 has Al_8_(OH)_10_ nodes.^21^ Some of these metal oxide clusters are more stable when they are present as MOF nodes rather than as the cores of carboxylate complexes, as illustrated, for example, by Al_3_O in MIL-100/MIL-101—these MOFs are both quite stable,^19,20^ but the metal oxide cluster requires bulky ligands to protect it as a carboxylate complex, as in [Al_3_(μ_3_-O)(μ-O_2_CCF_3_)_6_(THF)_3_][(Me_3_Si)_3_CAl(O_2_CCF_3_)_3_]·C_7_H_8_.^75^ In contrast, some metal oxo clusters are quite common as carboxylate complexes, but it is still challenging to connect them into MOFs;^76^ examples are the M_4_O_4_ cubic structure, Co_4_O_4_(CH_3_COO)_4_(C_5_H_5_N)_4_,^77,78^ and Mn_4_O_4_(O_2_P(Ph)_2_)_6_.^79,80^

We posit that the interplay between the chemistry of metal oxo compounds, especially metal oxo carboxylate complexes, and the chemistry of MOFs will continue to develop, to the benefit of both.

## OUTLOOK

The results summarized here show that important properties of metal oxide cluster-containing MOFs can be accounted for in the relationship between the degree of hydrolysis and the degree of substitution of the MOF nodes. We posit that recognition of the correlations presented here may help strengthen the connections between metal oxo cluster chemistry and MOF chemistry and may help in the design and synthesis of new, stable MOFs. The data suggest that stable MOFs should have *d*_c_ values between 1.0 and 1.33 and *d*_s_ values between 1.5 and 2.0 to achieve the highest stability (in air). We suggest this as a rule of thumb that might provide guidance for future development of stable MOFs and, further, that theory might be of value in predicting these values for MOFs that have not yet been made.
